# Natural Polymer-Based Hydrogel Platforms for Organoid and Microphysiological Systems: Mechanistic Insights and Translational Perspectives

**DOI:** 10.3390/polym17152109

**Published:** 2025-07-31

**Authors:** Yeonoh Cho, Jungmok You, Jong Hun Lee

**Affiliations:** 1 Department of Food Science and Biotechnology, Gachon University, 1342 Seongnam-daero, Seongnam-si 13120, Gyeonggi-do, Republic of Korea; 970907cyo@gachon.ac.kr; 2 Department of Convergent Biotechnology & Advanced Materials Science, BK21 Interdisciplinary Program in IT-Bio Convergence System, Kyung Hee University, Yongin-si 17104, Gyeonggi-do, Republic of Korea

**Keywords:** natural polymer hydrogel, organoid culture, microphysiological system, crosslinking strategy, biofunctionalization

## Abstract

Organoids and microphysiological systems (MPSs) have emerged as physiologically relevant platforms that recapitulate key structural and functional features of human organs, tissues, and microenvironments. As one of the essential components that define the success of these systems, hydrogels play the central role of providing a three-dimensional, biomimetic scaffold that supports cell viability, spatial organization, and dynamic signaling. Natural polymer-based hydrogels, derived from materials such as collagen, gelatin, hyaluronic acid, and alginate, offer favorable properties including biocompatibility, degradability, and an extracellular matrix-like architecture. This review presents recent advances in the design and application of such hydrogels, focusing on crosslinking strategies (physical, chemical, and hybrid), the viscoelastic characteristics, and stimuli-responsive behaviors. The influence of these materials on cellular processes, such as stemness maintenance, differentiation, and morphogenesis, is critically examined. Furthermore, the applications of organoid culture and dynamic MPS platforms are discussed, highlighting their roles in morphogen delivery, barrier formation, and vascularization. Current challenges and future perspectives toward achieving standardized, scalable, and translational hydrogel systems are also addressed.

## 1. Introduction

The emergence of three-dimensional (3D) cell culture platforms, particularly organoids and microphysiological systems (MPSs), has transformed in vitro tissue modeling by providing more physiologically relevant alternatives to conventional two-dimensional cultures. Organoids—self-organizing, stem cell-derived miniature organs—and MPSs, also known as “organ-on-a-chip” systems, enable the study of tissue development, disease mechanisms, drug efficacy, and toxicity with improved fidelity to human physiology [1]. Central to the success of these systems is the extracellular matrix (ECM)-like hydrogel scaffold, which supports tissue organization and morphogen gradients, and integrates mechanical cues [2,3].

Among the existing scaffolds, Matrigel, a basement membrane extract derived from mouse sarcoma, comprises a complex mixture of ECM proteins and growth factors and has been widely adopted for organoid culture due to its complex mixture of ECM proteins and growth factors [4]. However, Matrigel presents critical limitations, including an undefined composition, batch-to-batch variability, limited mechanical tunability, and xenogeneic origin, which hinder reproducibility and clinical translation [5]. These drawbacks necessitate the development of alternative hydrogel systems that are biochemically defined, tunable, and compatible with bioengineering workflows. Natural polymer-based hydrogels have emerged as attractive candidates for organoid and MPS scaffolding because of their inherent biocompatibility, biodegradability, and similarity to the native ECM [6]. Polymers such as collagen, hyaluronic acid (HA), gelatin, alginate, and chitosan provide cell-binding motifs and enzymatically cleavable domains, enabling dynamic cell–matrix interactions [7,8]. Moreover, these materials can be chemically modified or blended with synthetic polymers to tailor their stiffness, degradation rate, and viscoelastic behavior, which are critical for supporting stem cell differentiation and tissue morphogenesis [9,10].

Collagen- and HA-based hydrogels have yielded positive outcomes in modeling intestinal, hepatic, and neural organoids, whereas fibrin and gelatin scaffolds have facilitated vascularization and stromal integration in co-culture systems [11,12,13,14]. Natural hydrogels can mimic tissue-specific ECM environments, rendering them effective for use in MPS platforms where control of the spatial patterning, perfusion, and mechanical loading is essential [2,15].

As limitations, natural polymers suffer from mechanical weaknesses, uncontrolled degradation, and rapid gelation kinetics, which pose challenges for their reproducible fabrication and integration into complex devices. As prospective solutions to these issues, semi-synthetic hydrogels such as gelatin methacrylate (GelMA) and HA-methacrylate (HAMA) enable photopolymerization-based crosslinking and precise control over network architecture [16,17]. Interpenetrating polymer networks (IPNs) and click chemistry-based modular assemblies have been explored as platforms for bolstering the stability of these systems while preserving the biofunctionality [18].

Beyond static support, hydrogels actively regulate tissue processes by incorporating biochemical and physical cues. By tuning the mechanical properties, stiffness matching with specific tissues can be achieved, whereas the mesh size affects the molecular diffusion and growth factor gradients [19]. Precise control of cell adhesion, migration, and morphogenensis is attainable through functionalization with peptides, growth factor-binding motifs, and stimuli-responsive elements [20]. These capabilities are essential for recapitulating the complex signaling environments of disease development and tissue regeneration [21].

Advancements are evolving toward scalable, regulatory-compliant hydrogel platforms for clinical and industrial applications. Recombinant or chemically defined natural polymers have been explored as GMP-compatible alternatives to animal-derived matrices [22]. The demand for hydrogels that are compatible with bioprinting, microfluidic integration, and high-throughput screening workflows is growing [23].

In this review, we aim to provide a comprehensive design framework for next-generation hydrogel platforms that support reproducible, scalable, and physiologically relevant 3D tissue models. Particular focus is placed on natural polymer-based hydrogels, which have garnered significant attention in recent years for their dual role as scaffolds in organoid culture and as biofunctional matrices for providing an ECM-mimicking environment in MPSs. We summarize the structural and functional principles of these materials, including the classification of major natural polymers, their chemical modification strategies, and the physicochemical parameters that modulate cell behavior. By highlighting emerging application-specific use of hydrogel, this review seeks to guide the rational design of hydrogel systems tailored to both organoid and MPS platforms in the evolving landscape of advanced in vitro modeling.

## 2. Hydrogel Fundamentals for Organotypic Systems

### 2.1. Structural and Physical Principles

Natural polymer-based hydrogels, whether physically or chemically crosslinked, form three-dimensional (3D) networks that exhibit characteristic features such as swelling, mesh structure, and solute diffusivity. Chemically crosslinked natural polymer-based hydrogels, which represent one of the most commonly used crosslinking methods, form a three-dimensional (3D) network maintained by covalent bonds between polymer chains, offering enhanced mechanical stability and structural integrity. These crosslinks are formed via strategies such as small molecule crosslinkers (e.g., glutaraldehyde, succinic anhydride), photoinitiated polymerization, enzymatic catalysis, or interpenetrating polymer networks (IPNs) [24,25]. The crosslink density and network architecture critically influence the swelling behavior, mechanical strength, and overall stability of hydrogels, and can be tailored for applications ranging from tissue engineering to biosensing.

**Figure 1:**
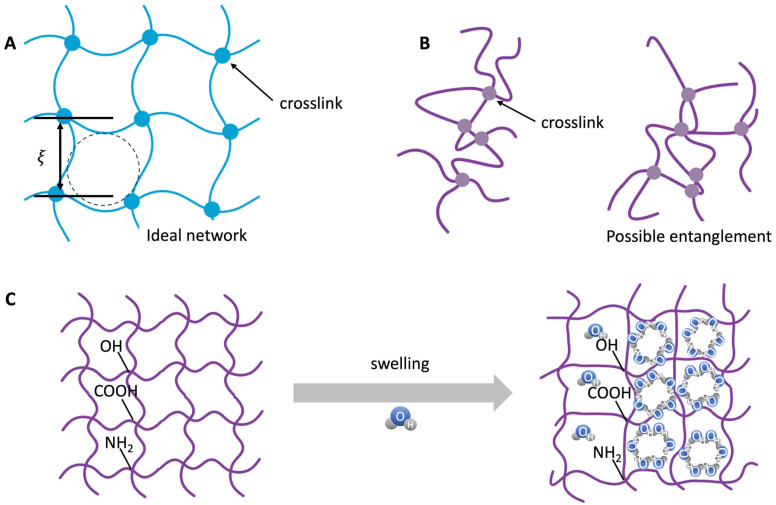
Schematic representation of mesh size in hydrogel networks and its functional implications. (**A**) In an ideal polymer network, mesh size (ξ) is uniform throughout the structure. The mesh size in the hydrogel network is depicted by a dashed line cycle. (**B**) In contrast, real hydrogels exhibit heterogeneous mesh size distributions due to variations in crosslinking density, polymer chain length, and chain entanglement. (**C**) Mesh size plays a critical role in governing the swelling behavior and mechanical integrity of hydrogels: larger mesh sizes permit greater water uptake during swelling but typically result in reduced mechanical strength. Additionally, ξ directly influences solute transport, with smaller meshes hindering the diffusion of large molecules and larger meshes facilitating enhanced molecular permeability.

The network serves as the fundamental structural framework, dictating the hydrogel’s mechanical, swelling, and transport properties [26,27]. Mesh size (ξ) is defined as the average distance between adjacent crosslinking points or structural constraints within the polymer network. While idealized networks assume a uniform mesh size, actual hydrogels typically exhibit heterogeneous distributions due to variations in crosslinking density, polymer chain length, and entanglement complexit, which are depicted in Figure 1A,B. Mesh size determines the degree of porosity and molecular diffusivity within the hydrogel matrix [25]. The significant swelling capacity of hydrogels is driven by their hydrophilic functional groups, such as –OH, –COOH, and –NH_2_, resulting from water uptake via osmotic pressure and hydrogen bonding. Swelling is thermodynamically governed by the balance between polymer–solvent mixing entropy and the elastic retraction forces of the network, as described by the Flory–Huggins and rubber elasticity models [28]. Because swelling leads to changes in the mesh size, it exerts a direct influence on both the mechanical stiffness and permeability of the hydrogel Figure 1C. Concurrently, diffusion within the hydrogel matrix is regulated by the relative rates of solvent penetration and polymer chain relaxation, leading to either Fickian or anomalous transport profiles [29]. These principles collectively enable the rational design of hydrogels with tunable physical characteristics—such as elasticity, permeability, and solute transport—tailored for specific applications in organoid culture, drug delivery, and MPSs.

Hydrogels display significant swelling capacity due to their hydrophilic functional groups (–OH, –COOH, –NH_2_), which promote water uptake through hydrogen bonding and osmotic pressure [30]. The swelling degree is governed by a thermodynamic balance between polymer–solvent mixing entropy and elastic retraction of the network, as articulated in the Flory–Huggins theory and rubber elasticity models [28,31]. Mesh size—the average spacing between crosslinks—is inversely related to crosslinking density, thereby controlling solute diffusivity and nutrient transport [32].

Mechanical properties of these hydrogels can be tuned from soft (~100 Pa, brain-mimicking) to stiff (~1 MPa, cartilage-like) by adjusting polymer concentration and crosslinking strategy [33,34]. While increased crosslink density enhances mechanical resilience, it often reduces swelling capacity—a design trade-off that must be balanced according to functional needs.

Water diffusion behavior in hydrogels is categorized as Fickian or non-Fickian (anomalous), depending on the relative rates of solvent diffusion and polymer relaxation [35]. These dynamics are modeled using Fick’s second law, Crank’s extension for swelling systems, or empirical power laws. Water within hydrogels is present in multiple states—bound (primary and secondary) and free water—each contributing differently to the material’s mechanical and diffusive properties [36,37].

Taken together, these principles provide a framework for designing hydrogels with customized swelling, stiffness, and permeability for advanced biological modeling systems such as organoids and MPSs.

### 2.2. Crosslinking Strategies

**Figure 2:**
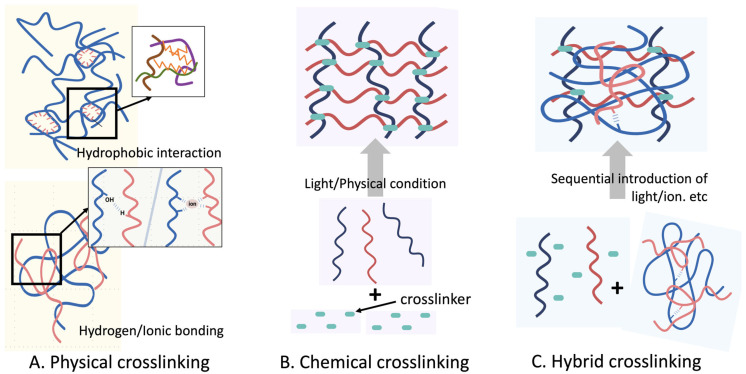
Comparison of physical, chemical, and hybrid crosslinking mechanisms in hydrogels. Schematic illustrating key characteristics of (**A**) physically crosslinked (non-covalent, reversible), (**B**) chemically crosslinked (covalent, irreversible), and (**C**) hybrid crosslinked hydrogels that integrate both mechanisms to balance dynamic responsiveness and mechanical stability.

The crosslinking strategy is a key determinant of the structural stability, physicochemical characteristics, and biological functionality of natural polymer-based hydrogels Figure 2. Crosslinking creates a network of interactions among the polymer chains, which controls the water retention, elasticity, porosity, and degradation kinetics of the hydrogels. Broadly, crosslinking methods are categorized as physical or chemical, each imparting distinct mechanical and biological properties to the resulting hydrogel. Furthermore, hybrid crosslinking approaches that aim to integrate the desirable features of both physical and chemical systems are being exploited for advanced biomedical applications [38,39,40].

### 2.3. Physical Crosslinking

Physical crosslinking involves the reversible association of polymer chains via non-covalent interactions such as hydrogen bonding, ionic bonding, hydrophobic interactions, crystallization, or chain entanglement Figure 2A. These interactions form thermally or ionically reversible networks that are typically formed under mild conditions, making them especially attractive for cell encapsulation and drug delivery applications where cytocompatibility is critical [41,42].

Ionic crosslinking of alginate with divalent cations (e.g., Ca^2+^, Sr^2+^) is widely employed in fabricating mechanically stable hydrogels that undergo gelation within seconds of mixing [43]. Similarly, gelatin and agarose undergo gelation via thermally induced chain aggregation and crystallization, allowing temperature-controlled formation and disassembly [44]. Although alginate ionic crosslinking involves interactions with divalent cations, it is classified as physical crosslinking because it relies on non-covalent ionic interactions rather than the formation of covalent bonds, which is a defining feature of chemical crosslinking. These physically crosslinked systems exhibit shear-thinning and self-healing behavior, which is advantageous for injectable or 3D-printable hydrogel platforms [45].

Physically crosslinked hydrogels generate soft, highly hydrated, and dynamic matrices that are particularly conducive to encapsulation of delicate cells and organoids. These hydrogels are typically characterized by low elastic moduli (10^1^–10^3^ Pa), mimicking the mechanical softness of tissues such as the brain, lung, or adipose tissue [46]. The reversible nature of the crosslinks in these hydrogels enables cell-mediated remodeling, expansion, and migration, which are essential for stem cell proliferation, organoid budding, and tissue morphogenesis [47].

However, the mechanical strength and long-term stability of physically crosslinked hydrogels are typically lower than those of chemically crosslinked hydrogels, limiting the application of the former in load-bearing tissue models. Although valuable in some contexts, the reversibility of physically crosslinked hydrogels may also lead to premature degradation or structural collapse under physiological conditions [48,49].

### 2.4. Chemical Crosslinking

Chemically crosslinked hydrogels are formed via covalent bonding between polymer chains, offering enhanced mechanical integrity, long-term structural stability, and resistance to rapid degradation Figure 2B. These characteristics render chemically crosslinked systems ideal for applications requiring prolonged culture, mechanical loading, or spatial fidelity, such as organoids or MPSs [50].

Chemical crosslinking can be triggered through various mechanisms such as small molecule crosslinking, photo-crosslinking, enzymatic crosslinking, and the formation of interpenetrating polymer networks. Small molecule crosslinkers such as glutaraldehyde, carbodiimides (e.g., EDC/NHS), and diacid chlorides form stable covalent bonds with functional groups such as amines, hydroxyls, and carboxyls. These methods are straightforward and widely used but often raise cytotoxicity concerns, particularly at high crosslinker concentrations [51,52]. Photo-crosslinking, which typically utilizes photo initiators such as Irgacure 2959 or LAP under UV or visible light, is applied to polymers bearing photoreactive groups such as methacrylate (e.g., gelatin methacrylate (GelMA) and hyaluronic acid methacrylate (HAMA)). This strategy enables rapid gelation under mild conditions and provides fine spatiotemporal control, allowing selective crosslinking within patterned geometries or defined regions in microengineered constructs [53,54,55]. Enzymatic crosslinking employs biocatalysts such as horseradish peroxidase (HRP), tyrosinase, or transglutaminase to mediate the formation of polymer networks under physiological conditions. These systems are particularly attractive as cell-laden hydrogels because of their gentle reaction conditions and compatibility with sensitive primary or stem cells [56,57]. The formation of interpenetrating polymer networks (IPNs) involves generating a secondary network within a pre-existing hydrogel matrix, which enhances the mechanical and biochemical properties by combining the functionalities of both systems [58,59].

Beyond the mechanical advantages, parameters such as the stiffness, porosity, swelling ratio, and mesh size of hydrogels can be precisely modulated by adjusting the degree of crosslinking (e.g., by varying the crosslinker concentration, duration of light exposure, or enzyme levels). These physicochemical properties directly affect cell–matrix interactions, including integrin binding, nutrient diffusion, and spatial confinement [60,61].

A higher crosslinking density typically leads to a smaller mesh size and stiffer gels, which can restrict cell spreading, migration, or organoid expansion. Conversely, lower crosslinking levels result in more malleable environments, favoring proliferation and lumen formation in epithelial organoids [62]. Importantly, the degradability of the hydrogel network, whether via enzymatic cleavage or hydrolytic lability, affects how cells dynamically remodel their surroundings.

The bioactivity and cytocompatibility of chemically crosslinked hydrogels are highly dependent on the crosslinking chemistry [63,64]. Although strong covalent bonds ensure durability, some chemicals, such as aldehydes or free radicals at high levels, may induce the formation of toxic byproducts or introduce residual reactivity, compromising cell viability [65]. In contrast, photo- and enzymatic crosslinking methods are often more biocompatible, especially when conducted under neutral pH, aqueous systems, and low-temperature conditions are employed.

Collectively, chemical crosslinking offers powerful control over the structure and function of hydrogels, enabling the rational design of tailored microenvironments for organoid culture, tissue modeling, and regenerative medicine. However, the crosslinking mechanisms and conditions must be carefully considered to balance structural requirements with biological performance.

### 2.5. Hybrid Crosslinking

To overcome the limitations associated with individual crosslinking methods, researchers have explored dual-crosslinked systems that incorporate both physical and chemical interactions Figure 2C. These hybrid hydrogels can synergistically combine the injectability, self-healing, and reversible behavior of physically crosslinked systems with the mechanical integrity and structural stability provided by chemical crosslinking, thereby expanding the functionality of hydrogels in advanced bioengineering applications such as organoid encapsulation and construction of MPSs [66].

By integrating covalent (GelMA) and ionic (alginate) crosslinking within hydrogel bioinks, Ana et al. precisely tuned the rheological, mechanical, and degradation properties, thereby enabling structurally stable 3D bioprinting. In a dual-printhead system, stripe-patterned constructs composed of GelMA–alginate and gelatin exhibited anisotropic mechanical behavior, highlighting the capacity of hybrid crosslinking for engineering directional stiffness [67].

Crosslinking of gelation–hyaluronic acid (HA) systems via the fast-forming Schiff base reaction of aldehyde-modified HA with amine-bearing gelatin, followed by secondary photopolymerization or enzymatic crosslinking to reinforce the mechanical stability and regulate the degradation profiles of the hydrogel. These sequential or simultaneous crosslinking strategies enable the construction of hydrogels with finely tunable viscoelasticity and degradation properties, which are especially important in long-term three-dimensional culture [68].

Importantly, hybrid crosslinking actively shapes the cellular microenvironment, thereby directly influencing organoid morphogenesis, polarization, and the maintenance of stemness. By integrating multiple crosslinking modes, such as ionic bond formation followed by covalent stabilization, mechanical stiffness can be decoupled from matrix degradability, enabling the recreation of complex, tissue-mimetic mechanical niches. Specifically, in the early stages, ionic crosslinks (e.g., Ca^2+^—alginate) produce a softer, more dynamic matrix that facilitates cell spreading, diffusion of nutrients, and nascent organoid formation; subsequent covalent crosslinking “locks in” these structures, preventing contraction and preserving the lumen architecture during prolonged culture [69].

Yunfei et al. developed a hybrid hydrogel matrix through the combined self-assembly of collagen with an oxidized hyaluronic acid–hydroxyapatite (OHAH) complex, which enabled the simultaneous formation of physical fibers and chemical crosslinking via a Schiff base reaction. Integrating physical self-assembly of collagen with Schiff base-mediated chemical crosslinking enabled tunable stress relaxation [70].

These finely engineered microenvironments facilitate advanced multicellular organization and dynamic remodeling, which are seldom achieved with single-mode crosslinked hydrogels. Overall, hybrid crosslinking strategies afford improved mechanical performance, while also crucially orchestrating cell–matrix signaling events that drive organoid development.

### 2.6. Biofunctionalization of Hydrogel

**Figure 3:**
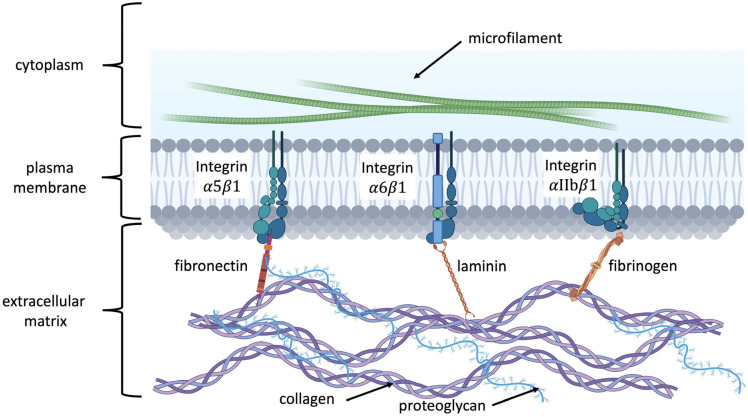
Integrin-mediated recognition of extracellular matrix (ECM) components. Specific integrin heterodimers bind to distinct ECM ligands: α5β1 and αvβ3 interact with fibronectin via its RGD motif; α6β1 recognizes laminin through its laminin G (LG) domains; and αIIbβ3 binds to fibrinogen during the coagulation process. The ECM consists of structural proteins such as collagen and proteoglycans, which not only provide mechanical scaffolding but also regulate cell adhesion and signaling pathways through integrin engagement.

Before discussing biofunctionalization, let us introduce the mechanism by which cells bind to the ECM to facilitate understanding Figure 3. Integrins are transmembrane receptors that mediate cell adhesion to the extracellular matrix (ECM) and link extracellular ligands to the intracellular actin cytoskeleton [71]. Specific integrin heterodimers recognize distinct ECM proteins: fibronectin via its RGD motif (e.g., α5β1, αvβ3), laminin through LG domains (e.g., α6β1), and fibrinogen during coagulation (e.g., αIIbβ3). The ECM is composed of structural proteins like collagen and proteoglycans, which provide mechanical support and regulate signaling. Upon ligand binding, integrin cytoplasmic tails recruit adaptor proteins such as talin and kindlin, which connect to actin filaments and initiate focal adhesion formation, enabling cells to sense and respond to their microenvironment [72,73].

Biofunctionalization refers to the strategic incorporation of biochemical signals into hydrogel matrices to regulate cellular behaviors such as adhesion, proliferation, and differentiation. Natural polymers such as collagen, gelatin, and hyaluronic acid inherently possess some cell-interactive domains; however, their functionality can be significantly enhanced by introducing the introduction of specific bioactive motifs, including peptides, proteins, and growth factor-binding moieties [74]. A comparative overview of key biofunctionalization strategies, Table 1, which summarizes representative biochemical modification strategies, such as the use of integrin-binding peptides, ECM-derived ligands, and nanomaterial composites, along with their respective effects on cellular behavior. Collectively, these modifications enable the creation of cell-instructive microenvironments that extend the functionality of hydrogel scaffolds beyond passive structural support.

In basic biofunctionalization, bioactive motifs such as RGD peptides are introduced into hydrogels. RGD peptides are short peptides comprising a tripeptide sequence of arginine–glycine–aspartic acid (Arg-Gly-Asp). This cell adhesion motif is recognized and bound by integrin receptors on the cell surface. RGD peptides enhance integrin-mediated adhesion, cell spreading, and cytoskeletal organization in inert scaffolds [75]. RGD-functionalized hydrogels can improve the cell density, migration, and formation of epithelial tissue, particularly when grafted onto polymers such as hyaluronic acid or gelatin [76].

**Table 1:** The expected effects of various relevant biofunctionalization factors.

Strategy	Examples	Effects	References
Integrin-specific peptides	GFOGER, YIGSR, IKVAV, REDV	Enhance cell adhesion, survival, and lineage-specific differentiation	[77,78,79,80]
ECM-derived biopolymers	HA + laminin, nanocellulose (NFC), chitosan	Mimic native ECM signals, promote proliferation and biocompatibility	[81,82,83,84]
Peptides + ions/metal cofactors	Osteostatin + Zn^2+^	Induce osteogenic markers (e.g., RUNX2, ALP), stimulate bone differentiation	[85,86,87,88]
Growth factor incorporation	BMP, FGF, VEGF, TGF-β	Improve cell survival, expansion, and lineage-specific tissue development	[89,90,91,92]
Synthetic polymer tuning	GelMA, PEG-4MAL (adjusted crosslinking/stiffness)	Tune cell–matrix interactions via viscoelastic and mechanical cues; support stemness and 3D structure	[93,94,95]
Nanomaterial composite systems	Laminin-coated nanofiber, graphene oxide, clay nanosheets	Guide neurite outgrowth, enhance mechanical integrity, enable bioelectronic applications	[96,97,98]

In addition to RGD peptides, various integrin-specific peptide motifs (e.g., GFOGER, YIGSR, and IKVAV), extracellular matrix-derived ligands (e.g., laminin, fibronectin, and collagen fragments), nanomaterial conjugates, and growth factor immobilization systems have been shown to significantly influence stem cell adhesion, viability, morphogenesis, and cell lineage commitment. These biofunctional signals can modulate integrin-mediated signaling, cytoskeletal organization, and local adhesion dynamics, which are essential for maintaining cell stemness and regulating specific differentiation pathways. Furthermore, introducing the nanostructural elements such as graphene oxide, clay nanosheets, or peptide-coated nanofibers into hydrogels further enhances their mechanical stiffness, electrical conductivity, and topographic guidance, which collectively exert synergistic effects on stem cell behavior. Similarly, spatially and temporally defined regulation of cell fate can be achieved by the immobilization or controlled release of bioactive molecules such as BMP-2, FGF-2, and VEGF. The expected effects of various relevant biofunctionalization factors are summarized in Table 1.

Finally, the properties of stimuli-responsive hydrogels change their properties in response to their surroundings. Stimuli-responsive hydrogels, particularly those responsive to pH or temperature, offer dynamic platforms for regulating cellular behavior and tissue microenvironments in organoids and MPSs.

pH-responsive hydrogels, typically composed of polymers containing weak acidic or weak basic moieties (e.g., poly(acrylic acid) and chitosan), undergo reversible swelling or deswelling in response to local pH fluctuations. This property enables the controlled release of bioactive molecules or on-demand matrix remodeling, which is particularly useful for mimicking the acidic environments of inflamed or tumor tissues [99,100]. Conversely, thermo-responsive hydrogels, such as those based on poly(N-isopropylacrylamide) (PNIPAAm), exhibit sol–gel transitions near physiological temperatures (around 32–37 °C), enabling injectable delivery and rapid in situ gelation. Such temperature-dependent behavior facilitates the minimally invasive administration and spatial confinement of encapsulated cells or organoids. When integrated with natural polymers such as gelatin or hyaluronic acid, these responsive systems can be tailored to provide environmental sensitivity, as well as enhanced biocompatibility and bioactivity [101,102,103].

Taken together, these biofunctionalization approaches transform natural polymer hydrogels into cell-instructive matrices that not only support structures but also actively guide organoid development and tissue regeneration. By integrating tunable mechanics and degradability, physiologically relevant microenvironments can be achieved for next-generation MPS and organoid platforms.

## 3. Applications in Organoid Systems

Organoids are self-assembled 3D structures derived from stem cells or progenitor cells that recapitulate key features of native tissues. To faithfully mimic in vivo development and function, organoid culture platforms must provide a biomimetic microenvironment that supports both the maintenance of stemness and the induction of spatially organized differentiation. Hydrogels, particularly those based on natural polymers, serve as extracellular matrix (ECM) analogs that can be finely tuned to meet these distinct demands [104]. Table 2 provides an overview of representative natural polymers used in hydrogel formation, including their crosslinking mechanisms, gelation behaviors, biofunctional properties, and compatible cell types.

### 3.1. Maintenance of Stemness

Natural polymer-based hydrogels incorporating ECM-derived ligands can effectively mimic the stem cell niche by providing integrin- and receptor-mediated cell-binding motifs. Laminin, collagen, and hyaluronic acid (HA) are the most studied natural polymers owing to their ability to support the adhesion, proliferation, and lineage-specific differentiation of stem cells.

Laminin is a heterotrimeric glycoprotein composed of α-, β-, and γ-chains, with multiple isoforms such as laminin-111, -511, and -521. Laminin-derived short peptides, including YIGSR, IKVAV, PDGSR, and RGD, play critical roles in mediating stem cell adhesion and directing differentiation by interacting with integrin receptors [130,131,132]. Notably, binding to α6β1 integrin facilitates the attachment and expansion of human pluripotent stem cells (hPSCs), neural progenitor cells, and cardiomyocytes to laminin-coated or peptide-functionalized surfaces [133,134,135].

The stiffness of hydrogels is a crucial regulator of the fate of stem cells in organoid cultures. Softer matrices, typically below ~500 Pa, limit cytoskeletal tension and reduce the nuclear translocation of mechanosensitive regulators such as YAP/TAZ, thereby maintaining stemness in intestinal and neural organoids. In contrast, stiffer environments tend to promote differentiation [132,136,137]. Dynamic hydrogels that enable reversible ligand binding or incorporate controlled-release systems for niche factors such as R-spondin 1, EGF, and Noggin, thereby providing sustained morphogen signaling, which supports long-term stem cell renewal. These designs more closely mimic the temporal presentation of in vivo niches and enhance the stability of undifferentiated stem cell populations [138,139].

Precise spatiotemporal control of matrix stiffness and ligand availability can be achieved with phototunable or enzyme-degradable hydrogels, thereby allowing the decoupling of stem cell proliferation from differentiation. DeForest et al. developed a programmable niche platform in which wavelength-specific photochemical reactions were exploited to independently control the photoconjugation of pendant ligands and photocleavage of crosslinks, enabling dynamic modulation of the 3D cellular microenvironment to study and direct evaluation of cell behavior [140].

Similarly, hydrogels incorporating MMP-sensitive (enzyme-cleavable) linkers can release tethered niche factors, including R-spondin 1, EGF, and Noggin, only in response to cell-secreted enzymes [141]. This mimics the controlled morphogen delivery observed in vivo and supports sustained stemness during organoid expansion [20].

### 3.2. Induction of Organoid Differentiation and Morphogenesis

Organoid differentiation and morphogenesis are governed by the biochemical composition, mechanical characteristics, and dynamic responsiveness of hydrogel scaffolds. Natural and synthetic matrices such as liver ECM [142], decellularized ECM (dECM) [143], fibrin [144], alginate [145], and GelMA–HA composites [146] support lineage specification and structural organization across diverse organoid types, including cholangiocyte, epithelial, neural, and glioblastoma models. Moreover, the viscoelastic and plastic remodeling behaviors of collagen-based scaffolds guide complex morphogenic processes such as budding [147], tubular fusion [148], and branching [149], whereas advanced in situ bioprinting techniques enable spatially confined control over epithelial polarity, axon guidance, and tissue segmentation [143]. Beyond these structural functions, hydrogels also serve as platforms for the precise, context-dependent delivery of morphogens such as BMP4, Wnt3a, and retinoic acid through matrix incorporation, affinity-mediated retention, or microfluidic perfusion. Consequently, hydrogels enable the spatiotemporal modulation of cell-fate decisions that are critical for reproducible tissue patterning [150,151].

Liver ECM-derived hydrogels provide tissue-specific biochemical cues that maintain cholangiocyte identity and support differentiation, highlighting the role of the ECM composition in directing the fate of organoids. Their defined and bioactive matrix environment of liver ECM-derived hydrogels facilitates organoid morphogenesis and enables dynamic culture formats, demonstrating how scaffold properties influence both structural organization and lineage specification [142]. Hydrogels derived from a decellularized extracellular matrix (dECM) retain native tissue-specific structural and biochemical signals, enabling them to guide cell behavior and support tissue remodeling. Their injectability and adaptability of dECM hydrogels make them well-suited for both in vitro organoid culture and in vivo regenerative applications, emphasizing the importance of native matrix cues in directing organoid morphogenesis and functional integration [143]. Similarly, fibrin-based hydrogels with RGD motifs and laminin-111 support the growth of epithelial organoids by providing essential adhesion cues and mechanical softness. Budding morphogenesis is driven by internal pressure and localized cell contractility, highlighting the effect of the composition and mechanics of the scaffold in regulating organoid differentiation and shape [144]. Alginate hydrogels provide a defined, xeno-free scaffold that reduces size variability and supports efficient neurogenesis and gliogenesis in spinal cord organoids, comparable to Matrigel. These hydrogels also enhance the specificity of neural fate by suppressing off-target marker expression, making them suitable for controlled differentiation and disease modeling in neural organoid systems [145]. In the context of tumor organoids, GelMA–HA-based biomimetic hydrogels preserve the key genetic and transcriptomic features of glioblastoma, while offering a tunable and consistent alternative to Matrigel. This tunability enables precise modeling of cancer biology and supports translational applications in precision medicine [146]. Collagen hydrogels capable of contraction can be used to fuse cystic intestinal organoids into macroscopic tubular structures that recapitulate in vivo-like epithelial organization, including villus-like luminal surfaces and crypt-like buds. This illustrates the effects of scaffold mechanics and spatial constraints in guiding large-scale morphogenesis and extending stem cell self-organization from microcysts to anatomically relevant tissue architectures [147]. Branch elongation in human mammary organoids is driven by collective cell-generated tension that plastically remodels the surrounding collagen, forming stable matrix cages. This nonlinear mechanical response of the scaffold provides directional cues for continued morphogenesis, illustrating the effects of dynamic ECM mechanics in regulating epithelial branching and tissue architecture [148]. By controlling the composition, geometry, and stiffness of the hydrogel in situ, the system directs morphogenetic processes such as cell polarity, axon guidance, and branching morphogenesis, highlighting the critical role of scaffold dynamics in organoid differentiation and tissue architecture [149].

In addition to supporting stem cell expansion, hydrogel scaffolds must enable lineage-specific differentiation and spatial organization to recapitulate defined tissue architectures. Rather than simply requiring stemness-maintaining signals, this process often necessitates the precise and context-dependent delivery of morphogens—such as BMP4, Wnt3a, and retinoic acid—in a temporally and concentration-regulated manner. Hydrogels offer a versatile platform for the controlled delivery of these bioactive cues through strategies such as direct incorporation into the hydrogel matrix, affinity-based sequestration of heparin-binding growth factors, and perfusion-based delivery via microfluidic systems. Using these methods, spatiotemporal modulation of morphogen exposure can be achieved, thereby supporting the consistent and directed specification of cell fate within developing organoids [150,151].

## 4. Applications in Microphysiological Systems (MPSs)

Organoid models are essential platforms for studying the development, function, and diseases affecting human organs in vitro, and have remarkable biological relevance. However, these models have limited ability to fully replicate native tissue environments owing to the absence of key features such as vascularization, immune components, and spatially organized multicellular structures. These limitations arise from the inherent constraints of static 3D cultures in which diverse developmental cues and intercellular interactions cannot be precisely coordinated [144,152]. To address these challenges, MPSs, also known as organ-on-a-chip platforms, have emerged as advanced in vitro models that integrate living cells, biomaterials, and microengineering to recreate dynamic tissue-level processes. MPSs provide a comprehensive and physiologically relevant framework for modeling organ function by enabling controlled co-culture, fluid flow, and mechanical stimulation. MPSs, device geometries, and fluid structures are typically designed using rigid bulk polymers such as polydimethylsiloxane (PDMS), which is the primary substrate material in on-chip long-term platforms [153].

Whereas the internal compartments that recreate tissue-specific microenvironments within MPSs are constructed using natural or semi-synthetic hydrogels that mimic key features of the ECM. Semi-synthetic polymers such as gelatin methacryloyl (GelMA) contain integrin-binding RGD motifs and support photocrosslinking, while polyethylene glycol diacrylate (PEGDA) and methacrylated hyaluronic acid (HAMA) offer highly tunable mechanical stiffness and biodegradability, and are compatible with photopatterning techniques [154]. Hydrogels are critical components of MPSs, providing a biomimetic three-dimensional matrix that supports organoid growth, differentiation, and functional tissue organization within these engineered systems [155,156].

Recent studies have explored the use of hydrogels in MPSs to create human-mimetic environments that can serve as alternatives to organoids and support stem or primary cell differentiation. In this review, focus is placed on the key properties of the hydrogels utilized in MPSs, including synthetic materials such as polyethylene glycol (PEG), and representative MPS platforms that incorporate natural polymer-based hydrogels are highlighted.

### 4.1. Roles of Hydrogel in MPSs

Hydrogel-based platforms play a critical role in mimicking the cellular microenvironment within MPSs by enabling simultaneous cell loading, adhesion, and the exchange of nutrients and waste under physiologically relevant shear stress. These dynamic processes can be monitored via real-time microscopic observation [157,158].

Microporous hydrogels, having interconnected 3D architectures, promote convective fluid flow, which improves nutrient distribution and waste removal within the scaffolds. This property is particularly advantageous for maintaining cell viability in thick or densely populated constructs. In modularly assembled hydrogel systems, predefined and interconnected microchannels are integrated to enable immediate perfusion. When combined with endothelial cells and under dynamic flow conditions, these perfusable networks facilitate cell adhesion, spreading, and vascular-like organization. These outcomes demonstrate the potential of microporous and microfluidically engineered hydrogels for developing vascularized 3D tissue constructs. The utility of hydrogels lies in their ability to form complex, tunable networks with well-defined physical properties [159,160,161].

Clancy et al. developed a microfluidic MPS platform in which PEG hydrogels were used to encapsulate U87 glioblastoma cells within membrane-capped PDMS wells, ensuring uniform cell distribution and mechanical support. Controlled solute delivery was achieved by generating a concentration gradient, generating the utility of hydrogels in maintaining 3D cell viability of cells and enabling assessment of the drug response under perfused conditions [162]. The cyclic 3D mechanical stimulation of MSCs was achieved using a chip-based MPS integrating PEG-NB hydrogels with deformable OSTE-PDMS membranes. The system afforded enhanced cell viability and promoted the formation of a contractile myofibroblast phenotype. This approach highlights the role of dynamic mechanical cues in guiding cell behavior within hydrogel-based MPS platforms [163]. Although the system is not a fully integrated MPS, it presents a bone marrow-inspired macroporous hydrogel platform with tunable mechanical and biochemical features for supporting the large-scale generation of MSC–ECM spheroids. Through liquid–liquid phase separation and enzymatically degradable crosslinking, the hydrogel promotes dynamic cell–matrix interactions, sustains stemness, and enables gentle cell harvesting, offering a promising foundation for future MPS development targeting the bone marrow or stem cell niches [164].

### 4.2. Hydrogels as Dynamic Mediators of Barrier and Vascular Functionality in MPSs

By employing hydrogels in MPSs, complex tissue interfaces and perfusion-dependent functions that are challenging to achieve in conventional 3D cultures can be replicated. By permitting selective molecular diffusion while separating cell populations, hydrogels can model semi-permeable barriers such as the gut epithelium, alveolar–capillary junction, and blood–brain barrier [165]. Their integration into the MPS forms tight junctions, and supports TEER (trans-endothelial electrical resistance) measurement, and dynamic modulation of barrier systems under flow, providing a physiologically relevant platform for studying barrier dysfunction and drug permeability [166,167].

Hydrogels facilitate the formation of perfusable, endothelialized vascular-like networks that mimic the native microvasculature. Such networks can be created through the self-assembly of endothelial cells or by pre-patterning microchannels within the hydrogel matrix. Under continuous laminar flow, they exhibit key endothelial functions such as nitric oxide production, immune cell interaction, and selective permeability, enabling studies of inflammation, immune response, and vascular remodeling in physiologically relevant models [168,169]. In advanced multi-organoid systems, hydrogels serve as analogs of the unifying ECM, thus allowing the integration of diverse tissue types within a shared microfluidic platform. By precisely tuning the composition and stiffness of hydrogels, a single device can support diverse organ-specific microenvironments, including those of hepatic, cardiac, testicular, and cerebral organoids. These hydrogels not only preserve tissue-specific phenotypes during prolonged culturing, but also allow synchronized perfusion and communication across compartments, enabling the investigation of systemic responses to pharmacological agents or pathological stimuli [170,171]. Moreover, the dynamic nature of hydrogel-based MPSs enables precise temporal and spatial control over signaling gradients and mechanical cues, which are essential for mimicking developmental processes and decisions determining the fate of stem cells. For example, cyclic deformation of hydrogel matrices or exposure to differential shear stress profiles can direct lineage specification or recapitulate aspects of morphogenesis that are otherwise absent in static culture systems [172,173].

These insights highlight the expanding utility of hydrogels in enhancing the functional complexity of MPSs. In addition to their passive structural roles, hydrogels actively contribute to shaping dynamic cellular microenvironments by supporting tissue interfaces, guiding vascular development, and enabling the realization of coordinated multicellular systems. Continued advances in hydrogel design and fabrication techniques are expected to further refine organ-level mimicry and strengthen the translational potential of MPSs for biomedical applications.

## 5. Conclusions

Natural polymer-based hydrogels have emerged as versatile and biologically relevant scaffolding platforms for the development of organoids and MPSs. Their intrinsic biocompatibility, bioactivity, and enzymatically tunable properties enable the recapitulation of native ECM features critical for supporting stem cell maintenance, lineage-specific differentiation, and complex tissue morphogenesis. Through diverse crosslinking strategies—ranging from physically reversible networks to chemically stabilized architectures and hybrid combinations—these hydrogels offer precise control over the structural and mechanical cues that shape the cellular microenvironment.

Recent advances in hydrogel design, including the incorporation of biofunctional peptides, growth factor-binding domains, and stimuli-responsive chemistries, have further enhanced the capacity of these materials to guide dynamic cell–matrix interactions. These functionalizations, coupled with emerging techniques such as photopatterning, bioprinting, and integration into microfluidics, allow hydrogels to serve not only as passive supports, and as active regulators of morphogen gradients, barrier formation, and tissue-level organization.

However, several challenges remain unsolved. Physically crosslinked hydrogels often lack sufficient mechanical stability for long-term or load-bearing applications, whereas chemically crosslinked systems may introduce cytotoxic residues or limit cellular remodeling unless carefully engineered. Hybrid crosslinking approaches, although promising, require further optimization to achieve scalability and regulatory compliance [5,6]. Moreover, their ability to fully recapitulate tissue-specific biochemical and mechanical microenvironments is still incomplete, and functionalization strategies for spatiotemporal control over signaling remain technically challenging. Furthermore, most naturally derived hydrogels are not yet compatible with GMP-compliant manufacturing, which hinders clinical translation.

The development of next-generation hydrogels can be advanced through the convergence of material science, stem cell biology, and microsystem engineering. Efforts to create fully defined, reproducible, and GMP-compatible natural polymer-based hydrogels are essential for bridging the gap between experimental models and clinical translation. By enabling tunable, integrative, and biologically instructive environments, these platforms are poised to accelerate advances in regenerative medicine, drug screening, and human-specific disease modeling [20].

## Figures and Tables

**Table 2:** Comparison of natural polymers used in hydrogel systems.

Hydrogel Type	Origin and Gelation Behavior	Biofunctional Features	Processing Advantages/Limitations	Representative Cell Types Used	References
Collagen-I	Natural ECM protein; gelates via thermoresponsive self-assembly at neutral pH and body temperature	Provides native cell-binding motifs; promotes adhesion, proliferation, migration	Enables gradient formation and versatile 3D architecture; batch variability and limited mechanical strength	HUVECs, Caco-2, NSCs, iPSCs, fibroblasts	[105,106,107]
Gelatin	Hydrolyzed form of collagen; reversible thermal gelation	Contains RGD motifs; supports multiple cell types	Chemically modifiable (e.g., GelMA); poor mechanical rigidity without crosslinking, enzymatically degradable	Cardiomyocytes, interstitial cells	[108,109,110,111]
Chitosan	Polysaccharide from crustacean shells; forms gel in acidic pH	Structural similarity to glycosaminoglycans; pH-responsive swelling	Requires chemical modification for mechanical tuning; limited solubility at neutral pH	Chondrocytes, NSCs, hMSCs	[110,112,113,114,115]
Fibrin	Protein derived from blood plasma; gelates via thrombin-induced polymerization	Promotes wound healing, angiogenesis, hemostasis	Gelation time and stiffness tunable by thrombin/Factor XIIIa concentration; sensitive to protease degradation	HUVECs, hMSCs, fibroblasts	[116,117,118,119]
Agarose	Marine-derived polysaccharide; forms gel via temperature change (thermoresponsive)	Biocompatible, mechanically robust	Non-biodegradable; lacks natural cell-binding motifs unless modified	HCT116, chondrocytes	[120,121,122]
Alginate	Seaweed-derived anionic polymer; gelation via ionic crosslinking with Ca^2+^	Inert, biocompatible; easy to modify for stiffness and porosity	Poor cell adhesion unless conjugated with adhesive ligands; acidification during gelation may harm cells	MCF-7, MSCs, F98	[123,124,125,126]
Hyaluronic acid (HA)	Non-sulfated glycosaminoglycan; can be chemically crosslinked (e.g., photoinitiated)	Supports cell migration and morphogenesis; endogenous in ECM	Enzymatically degradable; low adhesion without modification	NIH-3T3 fibroblasts, endothelial cells	[127,128,129]
